# Inheritance characteristics and potential of genomic prediction for pungency levels in F_1_ progeny of chili pepper (*Capsicum annuum*)

**DOI:** 10.1270/jsbbs.25011

**Published:** 2025-08-08

**Authors:** Nahed Ahmed, Kenichi Matsushima, Yui Kumanomido, Mariasilvia D’Andrea, Valentino Palombo, Shino Futatsuyama, Kazuhiro Nemoto, Fumiya Kondo

**Affiliations:** 1 Department of Science and Technology, Graduate School of Medicine, Science and Technology, Shinshu University, Minamiminowa, Nagano 399-4598, Japan; 2 Institute of Agriculture, Academic Assembly Faculty, Shinshu University, 8304 Minamiminowa, Nagano 399-4598, Japan; 3 Department of Agriculture, Graduate School of Science and Technology, Shinshu University, 8304 Minamiminowa, Nagano 399-4598, Japan; 4 Department of Agriculture, Environment and Food Sciences, University of Molise, via Francesco De Sanctis, snc, 86100, Campobasso, Italy; 5 Faculty of Agriculture, Shinshu University, Minamiminowa, Nagano 399-4598, Japan; 6 Graduate School of Agriculture, Kyoto University, Kitashirakawa-oiwakecho, Sakyo-Ku, Kyoto 606-8502, Japan; 7 Japan Society for the Promotion of Science, Kojimachi Business Center Building, 5-3-1 Kojimachi, Chiyoda-ku, Tokyo 102-0083, Japan

**Keywords:** chili pepper, crossbreeding, genomic prediction, heterosis, pungency, capsaicinoid, F_1_-hybrid

## Abstract

Pungency levels (capsaicinoid content) are critical traits influencing the quality and commercial value of chili peppers (*Capsicum annuum*). However, their complex inheritance patterns make controlling them challenging when crossing different progeny in current breeding programs. As a potential solution, we explored genomic prediction (GP) for crossing different progeny based solely on parental data. In this initial study, we assessed the feasibility of GP in 156 F_1_ accessions derived from 20 parents within 132 inbred *C. annuum* accessions. Capsaicinoid content (capsaicin, dihydrocapsaicin, and their total) was quantified using high-performance liquid chromatography. Inheritance analysis revealed that nearly half of the F_1_ accessions exhibited high-parent heterosis (F_1_ > higher parent), particularly in crosses between lower-pungency parents. We then performed GP for F_1_ accessions using 3,149 single nucleotide polymorphisms from inbred accessions. Among 11 models tested, GBLUP-GAUSS tended to show high accuracy, with predicted values showing a significant positive correlation (r = 0.770, *P* < 0.01) with observed capsaicinoid content (μg·gDW^–1^), although the involvement of heterosis in reducing accuracy was observed. These findings suggest that GP can effectively rank pungency levels among F_1_ progeny based solely on parental information, providing valuable insights for developing GP-based breeding strategies in chili pepper.

## Introduction

Chili pepper (*Capsicum annuum*) is a major Solanaceae crop, along with tomato (*Solanum lycopersicum*), eggplant (*S. melongena*), and potato (*S. tuberosum*). It is cultivated worldwide and is an economically crucial crop supporting both large-scale production and smallholder farming systems (Kantar *et al.* 2016). Chili peppers have various uses, not only as vegetables but also as spices, owing to the pungency traits unique to the *Capsicum* genus (Duranova *et al.* 2022). This pungency is attributed to chemical compounds known as capsaicinoids (Suzuki and Iwai 1984), which are synthesized mainly in the placental septum tissue of chili pepper fruits (Fujiwake *et al.* 1982). The amount of capsaicinoids determines the pungency level of chili pepper fruits, which is closely related to their utility (vegetable or spice) and quality as a spice. Therefore, capsaicinoid content is one of the crucial traits in chili pepper breeding. Additionally, capsaicinoids have recently attracted attention as functional ingredients due to their anti-inflammatory, antioxidant, antitumor, and weight-loss properties (Naves *et al.* 2019). Thus, efficient production of capsaicinoids has become necessary, particularly the development of highly pungent cultivars.

Capsaicinoid content is known to be a quantitative trait controlled by multiple genetic loci and is influenced by environmental factors such as temperature and soil moisture (Arce-Rodríguez and Ochoa-Alejo 2019). Several previous studies have identified qualitative genes, such as *Pun1* (*acyltransferase 3* (*AT3*)), *putative aminotransferase* (*pAMT*), *MYB31*, and *ketoacyl-ACP reductase* (*CaKR1*), which determine the presence or absence of pungency (Han *et al.* 2019, Koeda *et al.* 2019, Lang *et al.* 2009, Stewart *et al.* 2005). However, only a few genes related to pungency levels have been reported, despite numerous quantitative trait loci (QTLs) having been identified through QTL analyses and genome-wide association studies (GWAS) (Ben-Chaim *et al.* 2006, Blum *et al.* 2003, Han *et al.* 2018, Lee *et al.* 2016, McLeod *et al.* 2023, Yarnes *et al.* 2013). It is therefore considered that minor QTLs, which are difficult to detect in genetic analyses, may also influence capsaicinoid content (Nimmakayala *et al.* 2016). Due to the complexity of capsaicinoid inheritance patterns and the limited genetic insights and molecular breeding tools available, estimating pungency levels in the progeny of crosses based on parental traits is challenging. This difficulty represents a barrier to the development of new cultivars through F_1_ hybridization and pedigree breeding, the two main methods of chili pepper breeding.

**Fig. 1.**
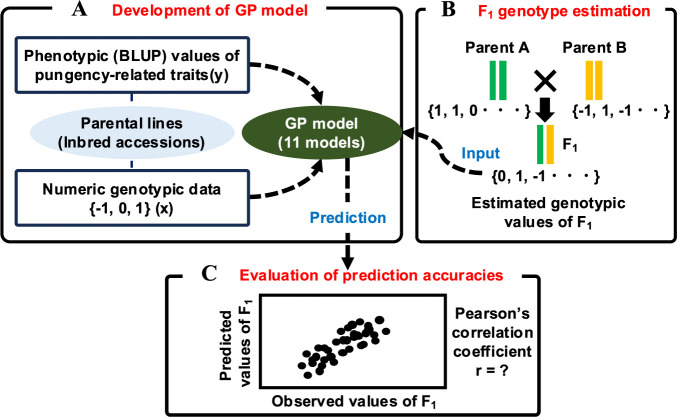
Schematic diagram of genomic prediction (GP) for each pungency-related trait of F_1_ progeny, based solely on parental information in this study. The workflow includes the development of GP models (11 types of models) using inbred accessions (parental lines) (A), the estimation of genotypic values for the F_1_ accessions (B), and the evaluation of GP accuracies through the correlation coefficient between observed and predicted phenotypic values. BLUP: best linear unbiased predictors.

Recently, genomic prediction (GP) has emerged as a promising solution to this challenge. GP is a powerful tool for predicting traits based on genome-wide marker genotypes, even without precise information on related QTL genotypes (Meuwissen *et al.* 2001). The utility of GP has been demonstrated in various crops (Crossa *et al.* 2017). Regarding capsaicinoid content in chili peppers, the potential of GP has been explored in a previous study (Kim *et al.* 2022), which demonstrated the feasibility of prediction at certain levels among inbred accessions. Based on these insights, GP might also be applicable for estimating capsaicinoid content when crossing different progeny using parental information. This approach begins with building a GP model based on the phenotypic and genotypic data for parental lines (Fig. 1a). The genotypes of the progeny are then estimated from their parental genotypic data (Fig. 1b). Finally, these estimated genotypes are used in the GP model to predict the phenotypes of the progeny (Fig. 1c). Similar approaches have been implemented in strawberries (*Fragaria* × *ananassa*) for several agricultural traits (Yamamoto *et al.* 2021), and our previous study revealed that this approach resulted in accurate predictions for five fruit-related traits (fruit length, width, shape index [length/width], weight, and pericarp thickness) (Kondo *et al.* 2025). These findings suggest the potential applicability of this method for predicting capsaicinoid content in chili peppers.

Before implementing the above GP approach in actual crossbreeding, two major points must be clarified. First, the inheritance characteristics and tendencies of capsaicinoid content in crossing progeny should be elucidated. Several studies have reported the existence of heterosis in F_1_ or F_2_ progeny with high heterozygosity (Arpaci *et al.* 2018, Naves *et al.* 2022, Rodríguez-Llanes *et al.* 2023), although consistent patterns and genetic factors remain unknown. Non-additive effects are known to influence GP accuracy (Varona *et al.* 2018), necessitating a deeper understanding of these effects. Second, it is important to clarify how GP works and how the characteristics that affect inheritance in crossing progeny affect GP performance. Chili peppers are generally self-pollinated plants with high homozygosity (Devi *et al.* 2021), which may present challenges for predicting traits in highly heterozygous populations. Understanding these two points is crucial for breeders to apply GP to pungency levels in chili peppers in crossbreeding scenarios. Therefore, we aimed to clarify these points and obtain empirical insights using F_1_ progeny.

In this study, we subjected 156 F_1_ accessions of chili peppers (*C. annuum*) derived from test crosses using 20 inbred accessions to investigate inheritance characteristics. We then performed GP using 132 inbred accessions, including the F_1_ parents. We evaluated prediction accuracies while exploring the effects of F_1_-specific inheritance characteristics and discussed the feasibility of using GP in crossbreeding for capsaicinoid content.

## Materials and Methods

### Plant materials and genotypic datasets

A total of 291 *Capsicum annuum* accessions from our previous study (Kondo *et al.* 2025) were used in this study. These comprised 132 inbred accessions and 159 F_1_ accessions. The inbred accessions included diverse commercial cultivars and genetic resources sourced from Asia and Central America, while the F_1_ accessions were generated through test crosses among 20 selected inbred lines. Detailed information regarding the plant materials, including derivations and parental combinations for the F_1_ accessions, is provided in our previous study (Kondo *et al.* 2025) and Supplemental Dataset 1. One plant per accession was cultivated at the Faculty of Agriculture, Shinshu University, Minamiminowa, Nagano, Japan, over three consecutive years (2021–2023). From each plant, ten fruits were harvested. To normalize the developmental stage of the fruit, we referenced fruit surface color and harvested only those beginning to change from deep green to brown. These fruit were used for the quantification of capsaicinoid content, as described below. Regarding the genotypic data, we used genome-wide single nucleotide polymorphisms (SNPs) data of inbred and F_1_ accessions obtained in our previous study (Kondo *et al.* 2025). Briefly, genomic DNA was extracted from young leaves of each accession, and multiplexed inter-simple sequence repeat genotyping by sequencing (MIG-seq) (Suyama and Matsuki 2015) was conducted. A total of 3,194 genome-wide SNPs were obtained, with a minor allele frequency greater than 0.05.

In our previous study (Kondo *et al.* 2025), their genetic characterization was conducted based on the 3,194 SNPs. The population structure of the 132 inbred accessions was characterized by the proportions of six distinct populations, which showed no clear stratification. The 20 parental accessions of the 156 F_1_ accessions also exhibited a diverse population structure among all inbred accessions. Based on this information, these accessions were considered genetically less biased plant materials. Additionally, the mean proportion of heterozygous loci in the F_1_ accessions was almost 2.5 times higher than in the inbred accessions, making them suitable for investigating the inheritance of pungency-related traits in high-heterozygosity populations and for evaluating GP based on the inbred accessions (a high-homozygosity population).

### Extraction and quantification of capsaicinoids

Capsaicinoid extraction was performed using placental tissue from harvested fruits, following previously established protocols (Kondo *et al.* 2021) with slight modifications. Ten placental septa per accession were lyophilized in the same zip-seal bag using a freeze-dryer (FDU-200; Tokyo Rikakikai Co., Ltd., Tokyo, Japan), and their total dry weights were recorded. The bulked tissues were ground into a fine powder using a mill mixer (YMB-401; Yamazen Corporation, Osaka, Japan). Capsaicinoids were extracted from 200 mg of powdered tissue using 8 mL of acetone, followed by a second extraction with 2 mL of ethyl acetate. The combined extracts were evaporated to dryness at 40°C and dissolved in 5 mL of methanol. Capsaicinoid content was quantified by high-performance liquid chromatography (HPLC). For analysis, 10 μL of the extract was filtered and analyzed by HPLC (LCsolution; Shimadzu Corporation, Kyoto, Japan) with a YMC-Pack ODS-A column (5 μm; 75 × 4.6 mm I.D.) coupled to a guard column (YMC-Guardpack ODS-A). The eluent consisted of methanol and distilled water (65:35) with 1% trifluoroacetic acid (TFA). The flow rate and temperature were set to 1.0 mL/min and 40°C, respectively. Detection was performed with a UV detector set to a wavelength of 280 nm. Capsaicin content (CAPgDW) and dihydrocapsaicin content (DCAPgDW) (μg·gDW^–1^) were quantified based on calibration curves using commercial standards (FUJIFILM Wako, Osaka, Japan). The total capsaicinoid content (TCAPgDW) (μg·gDW^–1^) was calculated as the sum of these two components. The dry weight of placental septum per fruit (DWP) was also calculated. Capsaicin, dihydrocapsaicin, and total capsaicinoid contents per fruit (CAPFL, DCAPFL, and TCAPFL, respectively) were then calculated by multiplying each content (μg·gDW^–1^) by DWP (μg/fruit).

### Phenotypic summarization of pungency-related traits

Representative values for seven pungency-related traits (DWP, CAPgDW, DCAPgDW, TCAPgDW, CAPFL, DCAPFL, TCAPFL) across the three cultivation years were calculated as best linear unbiased predictors (BLUPs) using the R package lme4 (Bates *et al.* 2015). Genotype (accession) and year were set to random and fixed effects, respectively, as described by Schmidt *et al.* (2019). Broad-sense heritability ($h_{b}^{2}$) for each trait was calculated as $h_{b}^{2}=\frac{\sigma_{g}^{2}}{\left(\sigma_{g}^{2}+\sigma_{\varepsilon }^{2}/n\right)}\times 100$, where $\sigma_{g}^{2}$ and $\sigma_{\varepsilon }^{2}$ represent genetic and residual variances, respectively, and *n* is the number of cultivation replicates (3 years). Using BLUP values, we calculated the mean, median, minimum, and maximum values for each trait to summarize their distributions. Pearson’s correlation coefficients were computed to evaluate relationships among the traits.

### Investigation of F_1_-dependent inheritance characteristics

To investigate the inheritance characteristics in F_1_ progeny, mid-parent (MP) and high-parent (HP) heterosis for each F_1_ accession were calculated following the method described by Ishimori *et al.* (2020):


$$\text{MP heterosis}(\%)=\left(\frac{{P_{F_{1}}-P}_{\text{mid}}}{P_{\text{mid}}}\right)\times 100$$

$$\text{HP heterosis}(\%)=\left(\frac{{P_{F_{1}}-P}_{\text{max}}}{P_{\text{max}}}\right)\times 100$$

where $P_{F_{1}}$ represents the BLUPs for the F_1_ progeny, $P_{\text{mid}}$ is the mean BLUP value of the two parents, and $P_{\text{max}}$ is the BLUP value for the parent with the higher phenotypic value.

Using the MP and HP heterosis values, we calculated the mean, median, minimum, and maximum values for each trait to summarize their distributions. For HP heterosis, the values were visualized as a heatmap on the parental combination matrix of the F_1_ accessions using the R package superheat (Barter and Yu 2017). We ignored phenotypic differences due to reciprocal crosses between parents. Cells in the matrix corresponding to actual crossing directions are marked with ‘R’, while cells for reversed crossing directions are marked with ‘F’ and display the same value as those marked with ‘R’. The F_1_ accessions were then classified into three groups based on their heterosis levels:

1. Non-heterosis (MP heterosis<0),

2. Medium-heterosis (MP heterosis>0 & HP heterosis<0), and

3. Strong heterosis (HP heterosis>0).

The additive effect (phenotypic differences between the two parents) for each F_1_ accession was calculated using the following formula described by Ukai (2002):


$$\text{Additive effect}=\frac{\left|P_{P1}-P_{P2}\right|}{2}$$

where $P_{P1}$ and $P_{P2}$ represent the phenotypic values (BLUPs) for the mother and father parents, respectively. The Pearson correlation coefficients between the additive effect and both MP and HP heterosis were also calculated.

Additionally, the relationship between MP and HP heterosis and the parental genetic distance was explored. The parental genetic distance was calculated as the Euclidean distance between the two parents in each F_1_ accession, based on numeric genotypic data (–1, 0, 1) from 3,194 SNPs. Pearson correlation coefficients between MP and HP heterosis and parental genetic distance were calculated to evaluate their relationships.

### Genomic prediction of pungency-related traits for F_1_ progeny

GP for seven pungency-related traits in F_1_ accessions was performed using models developed solely on inbred accessions, including the F_1_ parents. At first, GP models for each trait were constructed using BLUP data and numeric genotypic data (3,194 SNPs) for 132 inbred accessions (Fig. 1A). Eleven types of models were tested: Ridge, Least Absolute Shrinkage and Selection Operator (LASSO), Elastic Net (EN), Bayesian Ridge Regression (BRR), Bayes A, Bayes B, Bayes C, Genomic Best Linear Unbiased Prediction (GBLUP-A, GBLUP-AD, and GBLUP-GAUSS), and Random Forest (RF). Ridge, LASSO, and EN models were developed using the R package glmnet (Friedman *et al.* 2010). BRR, Bayes A, Bayes B, Bayes C, and RF models were constructed using the R package BWGS (Pérez-Rodríguez *et al.* 2013). GBLUP models utilized additive, dominance, and Gaussian kernel genetic relationship matrices, calculated using the R package RAINBOWR (Hamazaki and Iwata 2020), and the models were developed with the R package EMMREML (Akdemir *et al.* 2015). The genotypic values for F_1_ accessions ($g_{F_{1}}$) were calculated (Fig. 1b) as the mean of parental genotypic values: $g_{F_{1}}=\frac{g_{P1}+g_{P2}}{2}$, where $g_{P1}$ and $g_{P2}$ represent the numeric genotypic values (–1 for homozygous reference, 1 for homozygous alternate) for the mother and father parents, respectively. Heterozygous loci were assigned a value of 0. Segregation of heterozygous loci was not accounted for, and fractions (–0.5 and 0.5) resulting from heterozygous genotypes were adjusted to –1 and 1 for simplicity. The calculated $g_{F_{1}}$ values were finally input into the developed GP models, and phenotypic values for each F_1_ accession were predicted (Fig. 1c). Prediction accuracy was evaluated based on Pearson’s correlation coefficients between observed and predicted values.

### Analysis of the impact of F_1_-dependent inheritance characteristics on genomic prediction accuracy

To identify error factors affecting GP accuracy, the root square error (RSE) between observed and predicted values was initially calculated for each F_1_ accession. Then, four statistics related to F_1_-dependent inheritance (additive effects, MP heterosis (absolute value), HP heterosis (absolute value), and parental genetic distance) were used to compute Pearson’s correlation coefficients between RSE and these factors.

## Results

### Phenotypic summary of pungency-related traits

In the present study, seven pungency-related traits (DWP, CAPgDW, DCAPgDW, TCAPgDW, CAPFL, DCAPFL, and TCAPFL) were investigated in 291 chili pepper accessions, including inbred and F_1_ accessions, over three cultivation years. CAPFL, DCAPFL, and TCAPFL were calculated by multiplying the content per unit dry weight (μg·gDW^–1^) by the dry weight of the placental septum (gDW/fruit). In a previous study, it was reported that *C. annuum* hardly synthesizes capsaicinoids in the pericarp and seeds (Tanaka *et al.* 2017). We therefore assumed that capsaicinoids are synthesized only in the placental septum and calculated their contents per fruit accordingly. We calculated the BLUP values as the representative phenotypic values for each accession. The BLUP values for all traits showed significant positive correlations with raw phenotypic data from each cultivation year (Supplemental Fig. 1), confirming their validity as representative values. The BLUP values varied (Table 1), and broad-sense heritability ($h_{b}^{2}$) differed among traits (Table 1). Briefly, the medians were generally close to the means for most traits, and the mean contents of dihydrocapsaicin (both DCAPgDW and DCAPFL) were less than half of those of capsaicin. Notably, $h_{b}^{2}$ was highest in TCAPgDW ($h_{b}^{2}=94.0\%$) and lowest in DCAPFL ($h_{b}^{2}=77.0\%$). DWP also showed high heritability ($h_{b}^{2}=90.0\%$). Similar to TCAPgDW, CAPgDW and DCAPgDW (content per unit dry weight) exhibited higher heritability than their counterparts per fruit (TCAPFL, CAPFL, and DCAPFL).

**Fig. 2.**
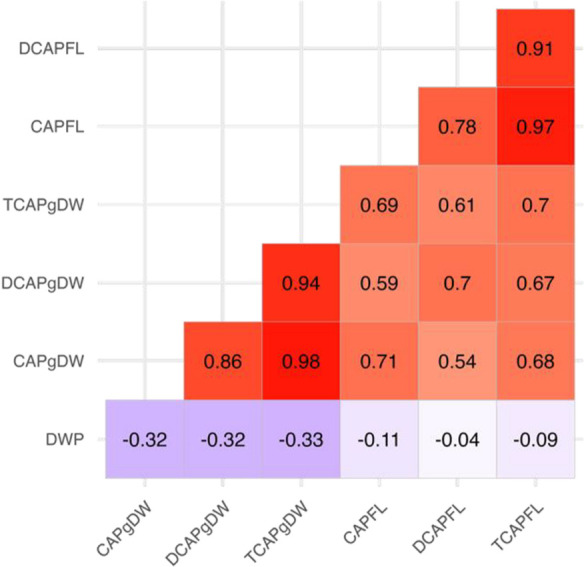
Pairwise correlations among pungency-related traits in all accessions (*n* = 291). Each box shows Pearson’s correlation coefficient between the best linear unbiased predictors (BLUP) values of two respective traits, represented with a color gradient ranging from red (positive correlation) to purple (negative correlation).

We also analyzed the relationships among BLUP values for the pungency-related traits. Pairwise correlation analysis revealed various inter-trait connections (Fig. 2). Specifically, TCAPgDW, CAPgDW, and DCAPgDW (content per unit dry weight) exhibited strong positive correlations with each other (r ≥ 0.86). Traits measured per fruit (e.g., TCAPFL, CAPFL, and DCAPFL) displayed a similar trend (r ≥ 0.78). However, correlations between content per unit dry weight and per fruit (e.g., TCAPgDW vs. TCAPFL) were relatively weaker (r ≤ 0.7). In contrast, DWP showed weak to moderate negative correlations with all other traits, ranging from r = –0.04 (DCAPFL) to r = –0.33 (TCAPgDW).

### F_1_-dependent inheritance characteristics of pungency-related traits

The inheritance characteristics of pungency-related traits in F_1_ accessions were assessed by evaluating MP and HP heterosis. Statistics for each trait are shown in Table 2. Notably, the means and medians of MP and HP heterosis were positive for most traits, except for DWP. This indicates that more than half of the F_1_ accessions exhibited higher phenotypic values than both MP and HP, although the opposite pattern was observed only in DWP. To further understand these patterns, F_1_ accessions were classified into three groups: non-heterosis (MP heterosis<0), medium heterosis (MP heterosis>0 & HP heterosis<0), and strong heterosis (HP heterosis>0). Their proportions were analyzed (Table 3). More than 70% of the F_1_ accessions exhibited heterosis (MP heterosis>0) for all traits except for DWP. Moreover, the proportion of accessions showing strong heterosis was 1.97–4.41 times higher than those showing medium heterosis. Conversely, only 30.8% of F_1_ accessions exhibited heterosis for DWP, and the proportion with strong heterosis was limited to 15.1%. These findings highlight the variability in heterosis levels across pungency-related traits.

**Fig. 3.**
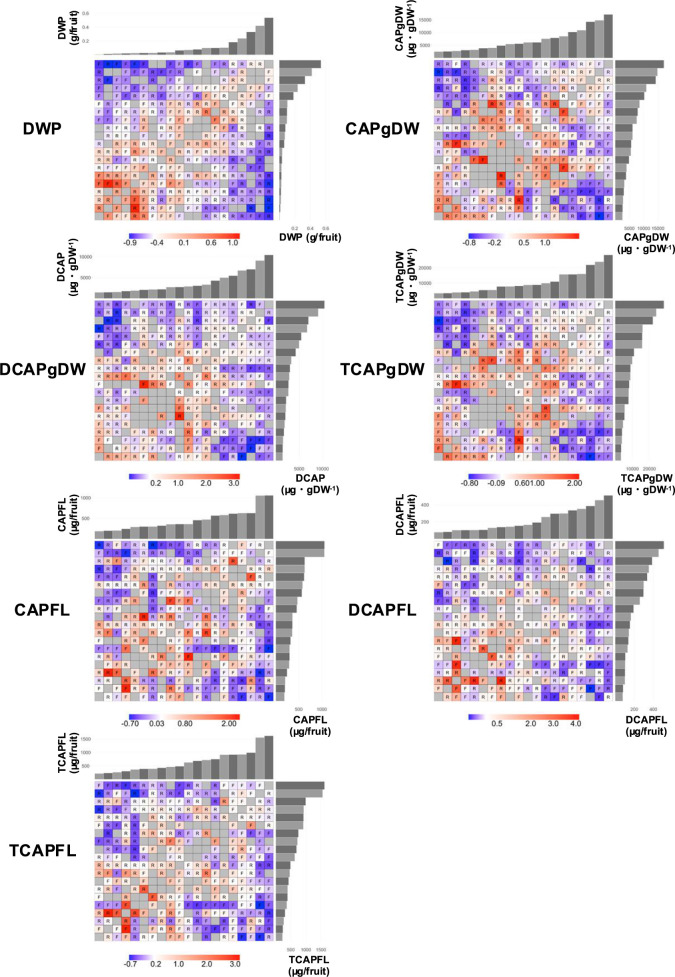
Heatmap of high-parent (HP) heterosis for the pungency-related traits in F_1_ accessions. Each panel represents a crossing combination table of F_1_ accessions, with rows indicating maternal parents (*n* = 20) and columns indicating paternal parents (*n* = 20). The color of each cell represents the magnitude of HP heterosis in the F_1_ progeny (dark red indicates a large positive value, while dark blue indicates a large negative value). In this analysis, phenotypic differences due to reciprocal crosses between parents were ignored. Thus, in this illustration, cells corresponding to actual crossing directions are marked with ‘R’, while cells for reversed crossing directions are marked with ‘F’ and display the same value as the former (R). Gray cells indicate no crossing was performed in either direction, and data are unavailable. The bar graphs at the top and right side of each panel represent the phenotypic values of the parental lines used in the crosses.

To identify factors associated with heterosis, we examined correlations between heterosis and other statistics. Additive effects, representing differences in phenotypic values between F_1_ parents, showed significant negative correlations with both MP and HP heterosis across all traits. This suggests that MP and HP heterosis tended to be smaller in F_1_ accessions whose parents exhibited larger phenotypic differences. In contrast, parental genetic distances, based on genotypic data from 3,194 SNPs, showed no significant correlation with either MP or HP heterosis across traits (Table 4). This indicates that genetic distance between parents was not consistently related to heterosis levels. Further, we investigated parental combinations and their influence on heterosis. Fig. 3 shows HP heterosis in F_1_ accessions, along with parental combinations and their phenotypic values. HP heterosis varied with parental combinations. Consistent with the correlation analysis, HP heterosis tended to be smaller in parental combinations with large phenotypic differences. Conversely, HP heterosis was larger in F_1_ accessions derived from parents with similar phenotypic values, especially when both parents exhibited lower phenotypic values.

### Accuracy of genomic prediction for F_1_ accessions

**Fig. 4.**
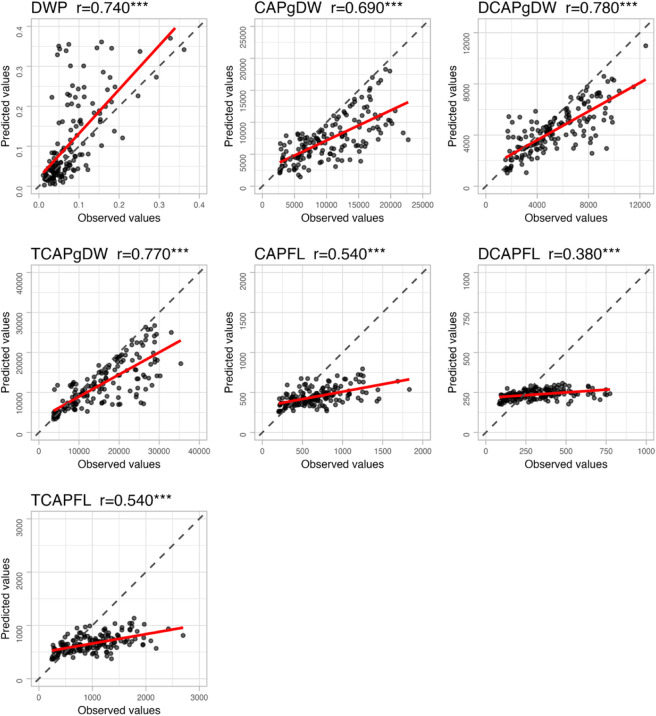
Accuracy of genomic prediction (GP) for pungency-related traits in F_1_ accessions (*n* = 159). The GP model was constructed using GBLUP-GAUSS, based solely on inbred accessions (parental lines, *n* = 132). The phenotypic values of the F_1_ accessions were predicted by inputting the estimated genotypic values of F_1_ individuals into the developed GP models. In each scatter plot, the red solid line represents the least squares regression line, while the black dotted line indicates the positions where predicted values match the observed values. r: Pearson’s correlation coefficient between observed and predicted phenotypic values. ***: significant at *P* < 0.001.

We performed genomic prediction (GP) for each pungency-related trait in F_1_ accessions (*n* = 159) using data from inbred accessions (*n* = 132), including F_1_ parents. We evaluated 11 GP models, and GP accuracies varied depending on the model and trait (Table 5). Among the models, GBLUP-GAUSS tended to show high accuracy regardless of the differences in traits, although this model did not always exhibit the best performance for all traits. Scatter plots of observed vs. predicted values with this model are shown in Fig. 4. GP accuracy was highest for TCAPgDW (r = 0.77) and lowest for DCAPFL (r = 0.38). Similar to the $h_{b}^{2}$ trends, TCAPgDW, CAPgDW, and DCAPgDW (content per unit dry weight) showed higher GP accuracy (r ≥ 0.68) than their per-fruit counterparts (e.g., CAPFL, DCAPFL, and TCAPFL). DWP also showed high accuracy (r = 0.74).

For all traits, significant positive correlations (*P* < 0.001) were observed between predicted and observed values (Fig. 4), indicating that the predicted rankings of phenotypic values closely matched actual rankings, although their levels varied by trait. When we focused on the GP error (differences between predicted and observed phenotypic values), some predicted values tended to be smaller than observed values for most traits, except for DWP. In particular, CAPFL, DCAPFL, and TCAPFL showed greater deviations, with predicted values exhibiting smaller variations than observed values. Conversely, some predicted DWP values were larger than observed values.

### Impact of F_1_-dependent inheritance characteristics on genomic prediction accuracy

To identify factors contributing to GP error, we analyzed correlations between root square error (RSE, predicted vs. observed values) and F_1_ inheritance characteristics (additive effects, MP heterosis, HP heterosis, and parental genetic distance) (Table 6). Both MP and HP heterosis showed significant positive correlations with RSE across all traits (*P* < 0.05), indicating that GP error tended to increase in F_1_ accessions with larger MP and HP heterosis. In contrast, additive effects and parental genetic distance showed no significant correlations with RSE for most traits, except for DWP. Specifically, for DWP, there was a strong positive correlation between additive effects and RSE (r = 0.82, *P* < 0.001), which was stronger than the correlation observed for MP and HP heterosis.

## Discussion

In the present study, we focused on the F_1_ generation of chili peppers, initially investigating the inheritance characteristics of pungency-related traits, and exploring the utility of GP for examining the relationship between GP error and inheritance characteristics.

As an initial insight into the inheritance of pungency-related traits, we found that capsaicinoid content per unit dry weight exhibited higher heritability than those per fruit (Table 1). This difference may be attributed to the calculation methods used for these values. In this study, capsaicinoid content per fruit was calculated by multiplying the content per unit dry weight by the dry weight of the placental septum. Since the additional involvement of placental septum size introduces an extra variable, its inheritance may be relatively lower than that of capsaicinoid content per unit dry weight. Consequently, capsaicinoid content per unit dry weight is regarded as a more practical measure for effective selection in chili pepper breeding.

Regarding the F_1_ progeny-dependent inheritance characteristics, we found many F_1_ accessions exhibited heterosis (MP heterosis>0 or HP heterosis>0) for capsaicinoid content, both per unit dry weight and per fruit (Table 3). Previous studies have reported heterosis in capsaicinoid content (Arpaci *et al.* 2018, Naves *et al.* 2022, Zewdie *et al.* 2001). However, the number of F_1_ accessions and parental combinations in these studies was limited compared to the present study, and the general trend of heterosis observed in our study had not been reported previously. Our results therefore suggest that heterosis is thus likely to occur in the pungency levels of chili peppers, providing valuable insights into F_1_ hybrid breeding of *C. annuum*. Conversely, dry weight of the placental septum (DWP) hardly exhibited heterosis (MP heterosis<0) (Table 3). In our previous study (Kondo *et al.* 2025), a similar trend was observed for fruit width and weight, where numerous F_1_ accessions exhibited negative dominance effects (${P_{F_{1}}-P}_{\text{mid}}<0$) for these traits. Given that the size or weight of the placental septum could influence fruit width and weight, these similarities are considered reasonable. We also observed a weak negative correlation between DWP and capsaicinoid content, both per unit dry weight and per fruit (Fig. 2), implying that an opposite heterosis pattern may occur.

Furthermore, we explored the parental combinations related to heterosis. We found that MP and HP heterosis tended to be smaller in F_1_ progenies derived from phenotypically distinct parents (Table 4), which was visually confirmed in the heatmap for HP heterosis (Fig. 3). Conversely, the heatmap also showed that combinations between phenotypically similar and lower parents exhibited larger HP heterosis. One possible explanation for these phenomena is the complementation effect of dominant alleles at different loci related to capsaicinoid biosynthesis or accumulation. When two low-pungency parents possessing recessive alleles at different loci are crossed, their F_1_ progeny may exhibit higher pungency than the parents due to complementation of dominant alleles from each parent. To better understand this mechanism, further studies will need to be conducted. On the other hand, no significant correlation was observed between MP and HP heterosis and parental genetic distance (Table 4), suggesting that hybrid vigor in *C. annuum* accessions is not always caused by genome-wide genetic differences between parents. Rather, it may be due to the complementarity of alleles at a few specific loci, as described above. Similar observations were reported by Geleta *et al.* (2004), who examined the relationship between heterosis levels of 15 fruit-related traits (excluding pungency traits) and genetic distance based on amplified restriction fragment length polymorphism (AFLP) markers in intraspecific F_1_ progenies of *C. annuum*. In contrast, several previous studies focusing on interspecific crosses (*C. annuum* × *C. chinense*) reported significant correlations between genetic distance and heterosis, suggesting that greater genetic divergence enhances hybrid vigor (Arpaci *et al.* 2018, Naves *et al.* 2022). These inconsistencies may result from differences in the scale of genetic diversity between interspecific and intraspecific crosses, warranting further analysis.

The present study performed GP for pungency-related traits in the F_1_ accessions using only the inbred accessions as the training population (Fig. 1). We found that GBLUP-GAUSS was a superior model, providing high GP accuracies despite the differences in traits. This model incorporates non-additive genetic effects, such as dominance and epistasis, by calculating Gaussian kernel genetic relationship matrices (Gianola *et al.* 2006, Gianola and van Kaam 2008). Several previous studies have demonstrated its superior performance in GP for F_1_ progenies of strawberries (*Fragaria* × *ananassa*), sorghum (*Sorghum bicolor*), and chili pepper (*C. annuum*) based on their parental data (Ishimori *et al.* 2020, Kondo *et al.* 2025, Yamamoto *et al.* 2021). Additionally, Kim *et al.* (2022) performed GP of capsaicinoid content within inbred lines and found that GBLUP-GAUSS (referred to as Reproducing Kernel Hilbert Spaces (RKHS) in their study) tended to show higher GP accuracies than other models examined. Given these findings, GBLUP-GAUSS appears to be a generally applicable model for GP across different populations and traits. We also found that capsaicinoid content per unit dry weight (CAPgDW, DCAPgDW, and TCAPgDW) and DWP showed high GP accuracies (r ≥ 0.690), whereas capsaicinoid contents per fruit had lower accuracies (r ≤ 0.54) (Fig. 4). This trend was similar to that observed for broad-sense heritability (Table 1), suggesting that low heritability negatively affected GP accuracy. This result also indicates that capsaicinoid content per unit dry weight is preferable for GP to achieve high prediction accuracy. Although there were differences in GP accuracies, significant positive correlations were observed between predicted and observed phenotypic values (Fig. 4). This suggests that GP can effectively rank the phenotypic values of pungency-related traits in F_1_ accessions to a certain extent. However, further evaluation is needed to determine whether the accuracy is sufficient for specific breeding programs.

Despite the success of the present GP approach, its limitations were also evident. Specifically, some predicted phenotypic values tended to deviate from the observed values for most traits, with the largest discrepancies observed in CAPFL, DCAPFL, and TCAPFL (Fig. 4). Correlation analysis revealed that MP and HP heterosis were at least partially associated with these errors (Table 6). One possible explanation is that the inbred accessions used as the training population for GP had high homozygosity. As mentioned above, we previously reported that inbred accessions had less than half the heterozygosity observed in F_1_ accessions (Kondo *et al.* 2025). Consequently, it may be difficult to accurately predict the dominance effects emerging in F_1_ progenies using a GP model trained exclusively on a highly homozygous population. To overcome this limitation, incorporating some F_1_ accessions or commercial F_1_ varieties with high heterozygosity into the training population may be necessary. Further optimization of the training population will be required.

The present study comprehensively clarified the inheritance characteristics of pungency-related traits in F_1_ progenies. Additionally, it provided insights into the utility and limitations of the GP approach for predicting these traits based on parental information. Our findings suggest that the application of GP for assessing pungency-related traits in high-heterozygosity populations may be feasible using data from highly homozygous populations. Thus, evaluating GP performance not only in F_1_ progeny but also in other segregating generations, such as F_2_ progeny, is considered valuable. Finally, the empirical data presented in this study will serve as fundamental information for establishing GP-based breeding strategies, including F_1_ hybrid breeding and pedigree breeding.

## Author Contribution Statement

K.M. and F.K. respectively contributed to the conception and design of the study with the agreement of all authors. K.M. and F.K. prepared the plant materials used in the present study. Y.K. and S.H. contributed to harvesting fruit samples and extracting placental septum samples for capsaicinoid extraction. N.A. carried out the extraction and quantification of capsaicinoids. Phenotypic statistical analyses were mainly conducted by N.A. and F.K., while genomic prediction and its accuracy evaluation were carried out by N.A., M.D., V.P., and F.K. The first draft was prepared by N.A., F.K., and K.M., with all authors providing feedback on subsequent versions. All authors read and approved the final manuscript.

## Acknowledgments

The authors thank Mr. Shinsaku Murayama (Agricultural Co. Peppers.jp, Gunma, Japan) for providing plant materials, and Dr. Motoyuki Ishimori (Graduate School of Agricultural and Life Sciences, The University of Tokyo, Tokyo, Japan) for his theoretical and technical advice on GP. Computations were partially carried out on the NIG supercomputer at ROIS National Institute of Genetics. This research was financially supported by Yawataya Isogoro Inc. and the Japan Society for the Promotion of Science (JSPS) KAKENHI Grant Number 22J13069.

## Supplementary Material

Supplemental Figure

Supplemental Dataset

## Figures and Tables

**Table 1.** Phenotypic summary of the pungency-related traits in all accessions (*n* = 291)

Trait	Statistics of phenotypic (BLUP*^a^*) values				*h_b_*^2^ (%)*^b^*
	Mean ± SD	Median	Maximum	Minimum	
DWP (g/fruit)	0.097 ± 0.2	0.060	2.350	0.010	90.0
CAPgDW (μg·DW^–1^)	9,240 ± 5,681	8,007	29,833	2,536	90.0
DCAPgDW (μg·DW^–1^)	4,907 ± 2,819	4,409	15,403	1,468	83.0
TCAPgDW (μg·DW^–1^)	14,147 ± 8,733	12,582	44,963	3,381	94.0
CAPFL (μg/fruit)	604 ± 320	546	1,832	189	79.0
DCAPFL (μg/fruit)	296 ± 171	265	1,329	79	77.0
TCAPFL (μg/fruit)	899 ± 502	806	3,158	220	82.0

*^a^* Best linear unbiased prediction. *^b^* Broad-sense heritability described by Schmidt *et al.* (2019).

**Table 2.** Summary of mid-parent (MP) and high-parent (HP) heterosis (%)*^a^* in the F_1_ accessions (*n* = 159) for the pungency-related traits

Heterosis type	Trait	Statistics of MP and HP heterosis			
		Mean ± SD	Median	Maximum	Minimum
MP heterosis	DWP	–11.7 ± 45.5	19.5	189.6	–83.5
	CAPgDW	49.3 ± 66.7	39.1	264.9	–68.4
	DCAPgDW	45.6 ± 73.5	24.7	379.3	–59.3
	TCAPgDW	51.6 ± 71.5	36.1	320.1	–69.4
	CAPFL	51.7 ± 67.1	46.4	261.9	–57.3
	DCAPFL	57.2 ± 90.6	34.0	491.7	–64.4
	TCAPFL	59.2 ± 77.5	48.4	371.3	–58.3
HP heterosis	DWP	–37.2 ± 37.2	–47.4	113.4	–91.5
	CAPgDW	16.4 ± 56.4	8.2	181.6	–81.5
	DCAPgDW	14.6 ± 65.8	2.1	321.1	–75.4
	TCAPgDW	16.8 ± 59.9	6.2	211.7	–82.5
	CAPFL	22.3 ± 60.7	14.4	229.8	–72.4
	DCAPFL	23.4 ± 82.3	1.0	415.4	–76.4
	TCAPFL	24.5 ± 68.9	14.4	317.1	–74.4

*^a^* MP and HP heterosis described by Ishimori *et al.* (2020).

**Table 3.** Proportion of the F_1_ accessions (*n* = 159) classified into non-, medium-, and strong-heterosis groups*^a^*

Trait	Proportion (%)		
	Non-heterosis	Medium heterosis	Strong-heteorsis
DWP	69.2	15.7	15.1
CAPgDW	28.3	15.1	56.6
DCAPgDW	28.9	23.9	47.2
TCAPgDW	27.0	18.9	54.1
CAPFL	25.2	13.8	61.0
DCAPFL	28.3	20.8	50.9
TCAPFL	25.2	13.8	61.0

*^a^* Three groups based on the mid-parent (MP) and high-parent (HP) heterosis in the F_1_ accessions as follows: 1. Non-heterosis: 1. Non-heterosis (MP heterosis < 0), 2. Medium heterosis (MP heterosis > 0 & HP heterosis < 0), and 3. Strong-heterosis (HP heterosis > 0).

**Table 4.** Correlation of mid-parent (MP) and high-parent (HP) heterosis with additive effects and parental genetic distance

Trait	Additive effect*^a^*			Parental genetic distance*^b^*	
	MP heterosis	HP heterosis		MP heterosis	HP heterosis
DWP	–0.569^c ^***^d^	–0.631***		0.053^N.S ^*^e^*	0.047^N.S^
CAPgDW	–0.291***	–0.499***		–0.078^N.S^	0.073^N.S^
DCAPgDW	–0.353***	–0.514***		–0.104^N.S^	0.089^N.S^
TCAPgDW	–0.325***	–0.520***		0.088^N.S^	0.080^N.S^
CAPFL	–0.321***	–0.506***		–0.006^N.S^	0.013^N.S^
DCAPFL	–0.300***	–0.459***		–0.024^N.S^	0.010^N.S^
TCAPFL	–0.321***	–0.489***		–0.011^N.S^	0.021^N.S^

*^a^* Additive effect described by Ukai (2002). *^b^* Euclidean distance between the two parents in each F_1_ accession, based on numeric genotypic data (–1, 0, 1) derived from 3,194 SNPs. *^c^* Pearson’s correlation coefficient. *^d^* ***: Significance levels: *P* < 0.001. *^e^* No significant.

**Table 5.** Genomic prediction (GP) accuracy for seven pungency-related traits in the F_1_ accessions, using 11 models

Trait	GP Models											
	Ridge	LASSO	EN	GBLUP-GAUSS	GBLUP-A	GBLUP-AD	RF	Bayes A	Bayes B	Bayes C	BRR	Mean
DWP	0.68*^a^*	0.63	0.66	0.74	0.69	0.52	0.75	0.74	0.73	0.72	0.71	0.69
CAPgDW	0.63	0.54	0.55	0.68	0.67	0.67	0.63	0.66	0.61	0.66	0.66	0.63
DCAPgDW	0.55	0.54	0.57	0.78	0.59	0.59	0.49	0.58	0.56	0.57	0.57	0.58
TCAPgDW	0.63	0.53	0.55	0.77	0.67	0.67	0.58	0.65	0.64	0.66	0.67	0.64
CAPFL	0.5	0.15	–*^b^*	0.54	0.49	0.49	0.55	0.53	0.51	0.53	0.53	0.48
DCAPFL	0.31	0.15	–	0.38	0.33	0.33	0.34	0.41	0.42	0.4	0.4	0.35
TCAPFL	0.48	–	–	0.54	0.49	0.49	0.52	0.52	0.53	0.53	0.53	0.51

*^a^* Pearson’s correlation coefficient between observed and predicted values. *^b^* Correlation coefficient was not calculated because the predicted values were constant values in all F_1_ accessions.

**Table 6.** Correlations between root square error (RSE)*^a^* and F_1_-dependent inheritance characteristics

Trait	Correlation with root square error (RSE)			
	Additive effect	MP heteorsis (Absolute value)	HP heterosis (Absolute value)	Parental genetic distance
DWP	0.82^b ^***^c^	0.34***	0.50***	0.17*
CAPgDW	0.01^N.S ^*^d^*	0.67***	0.45***	0.07^N.S^
DCAPgDW	0.17^N.S^	0.24**	0.23*	0.12^N.S^
TCAPgDW	0.05^N.S^	0.51***	0.35***	0.02^N.S^
CAPFL	0.02^N.S^	0.76***	0.58***	0.04^N.S^
DCAPFL	0.01^N.S^	0.65***	0.47***	–0.05^N.S^
TCAPFL	0.02^N.S^	0.67***	0.48***	0.02^N.S^

*^a^* RSE between observed and predicted phenotypic values in the F_1_ accessions. Predicted phenotypic values were obtained genomic prediction with GBLUP-GAUSS model. *^b^* Pearson’s correlation coefficient. *^c^* *, **, ***: Significance levels: *P* < 0.05, *P* < 0.01, *P* < 0.001, respectively. *^d^* No significant.
