# Supplementary material for: Inheritance characteristics and potential of genomic prediction for pungency levels in F1 progeny of chili pepper (Capsicum annuum)
Source: Breed Sci. 2025 Aug 8;75(4):303–14. doi: 10.1270/jsbbs.25011 (PMC13051630; doi:10.1270/jsbbs.25011)
Supplement: Supplementary file 1 — Supplemental Figure [file 75_303_s1.pdf]

doi: 10.1270/jsbbs.25011

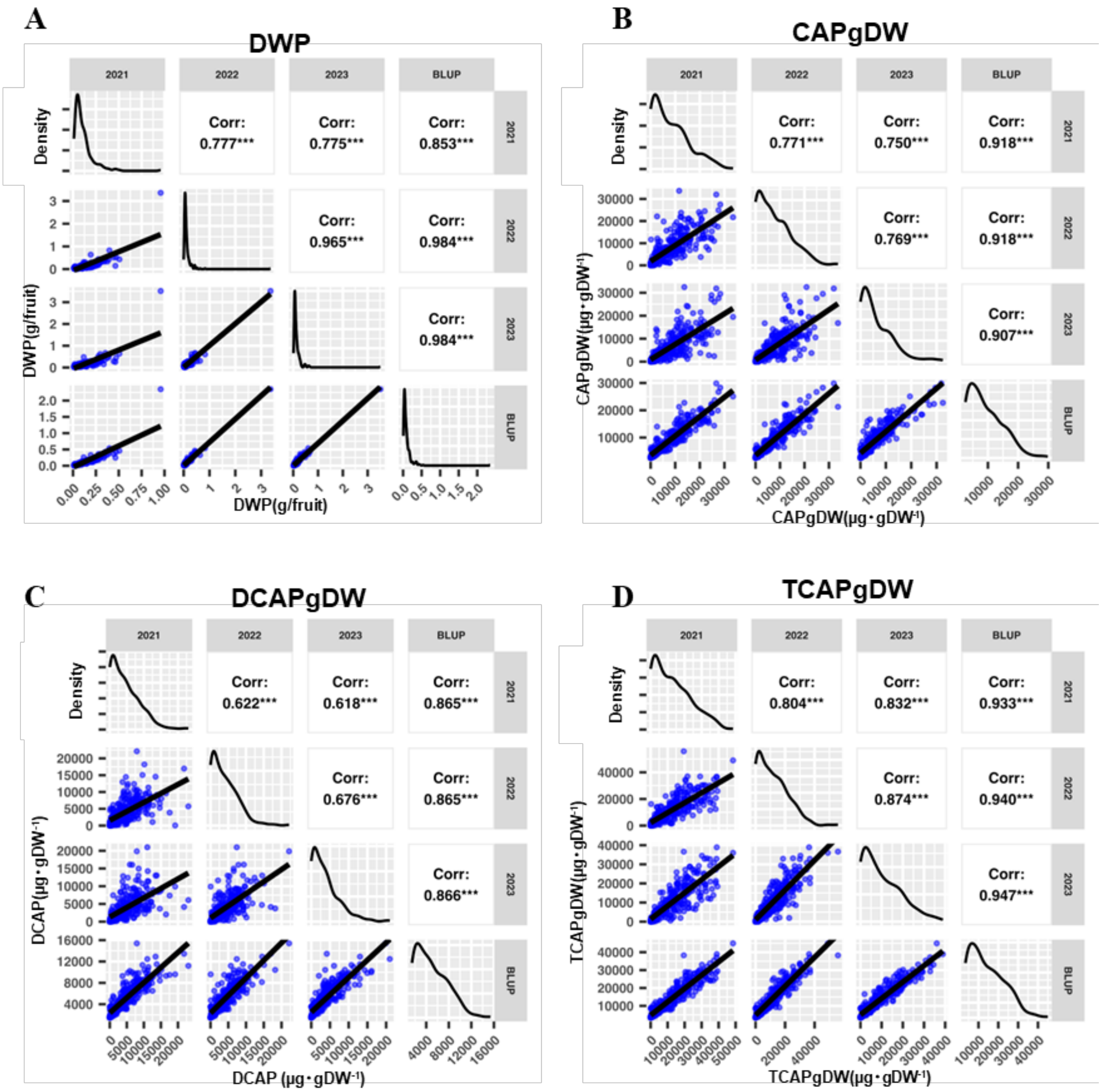

Supplemental Fig. 1. Relationship between best linear unbiased predictors (BLUP) values and actual phenotypic values for pungency-related traits across all accessions in each cultivation year (2021–2023). **(A)** The dry weight of placental septum per fruit (DWP). **(B)** Capsaicin content per unit dry weight (CAPgDW). **(C)** dihydrocapsaicin content unit dry weight (DCAPgDW). **(D)** Total capsaicinoid content per unit dry weight (TCAPgDW). Corr: Pearson's correlation coefficients. ***: significant at $P < 0.001$.

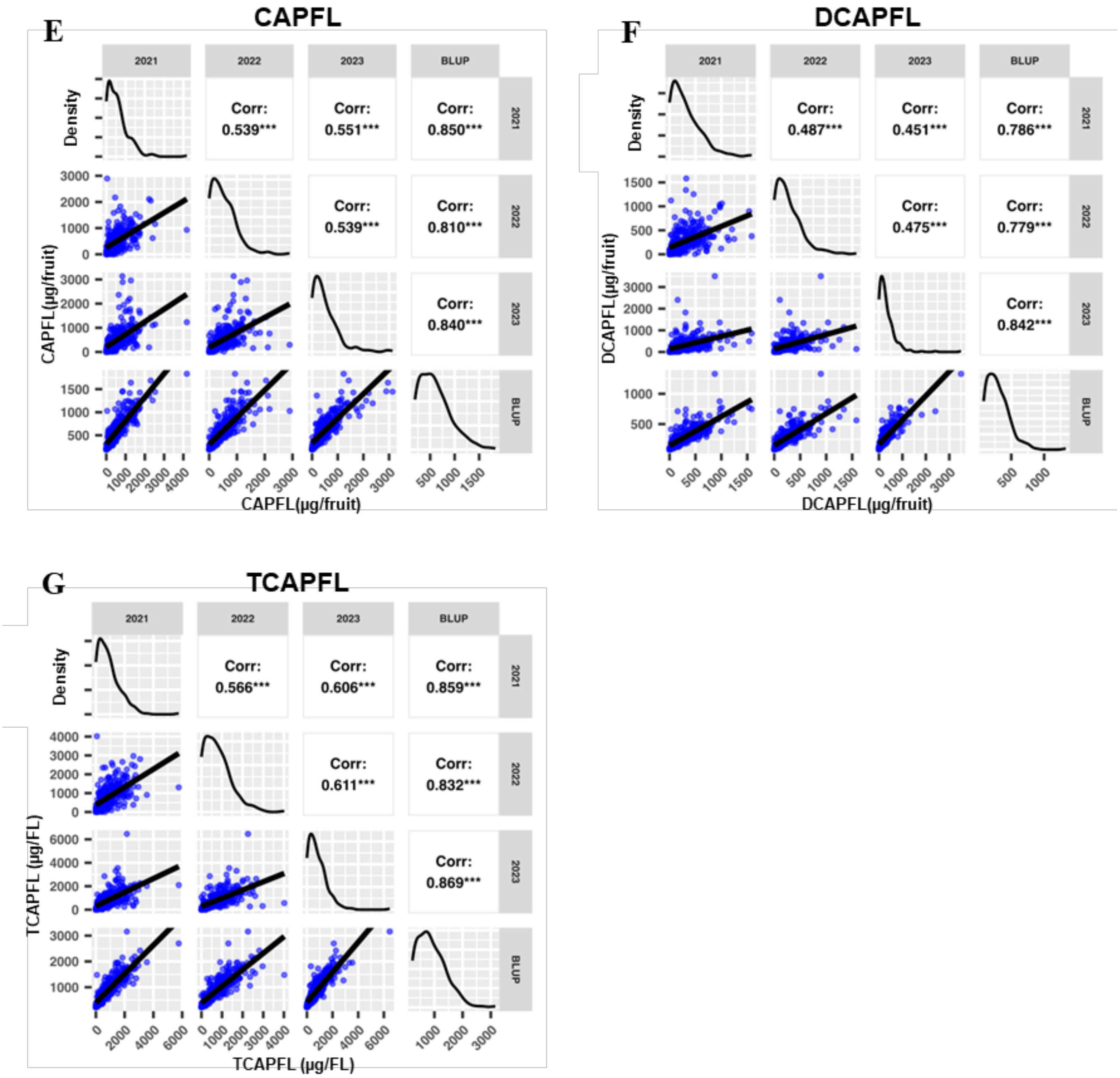

Supplemental Fig. 1. Continued. **(E)** Capsaicin content per fruit (CAPFL). **(F)** dihydrocapsaicin content per fruit (DCAFL). **(G)** Total capsaicinoid content per fruit (TCAPFL). Corr: Pearson's correlation coefficients. ***: significant at $P < 0.001$.
